# An investigation of fungal contamination on the surface of medicinal herbs in China

**DOI:** 10.1186/s13020-016-0124-7

**Published:** 2017-01-03

**Authors:** Run-sheng Zheng, Wen-li Wang, Jing Tan, Hui Xu, Ruo-ting Zhan, Wei-wen Chen

**Affiliations:** 1 Research Centre of Chinese Herbal Resource Science and Engineering, Guangzhou University of Chinese Medicine, Guangzhou, China; 2 Key Laboratory of Chinese Medicinal Resource from Lingnan, Ministry of Education, Guangzhou University of Chinese Medicine, Guangzhou, China

## Abstract

**Background:**

The dried parts of medicinal herbs are susceptible to the infection of fungi during pre- or post-harvest procedure. This study aimed to investigate the presence of fungi and their metabolites mycotoxins on the surface of medicinal herbs collected from China.

**Methods:**

Forty-five retail samples of 15 different medicinal herbs were collected from 3 different regions in China. Then the potential fungi were immediately washed off from the surface of each sample with 0.1% Tween-20 followed by incubation of the rinse on petri-dish with potato dextrose agar containing chloramphenicol at 28 °C. The obtained fungi were isolated as single colonies and then characterized by morphology and molecular identification using internal transcribed spacer (ITS) sequencing with extracted DNA. Meanwhile, the mycotoxin-producing potential of the isolates was studied by liquid chromatography-tandem mass spectrometry (LC-MS/MS).

**Results:**

A total of 126 fungi were identified from the surface of samples by morphology and ITS sequencing, with *Aspergillus* and *Penicillium* genera as the predominant contaminants. The mycotoxin-producing potential analysis showed that 6 of 8 *A. versicolor* isolates could produce sterigmatocystin. All 3 *A. aculeatus* isolates produced ochratoxin A, but only 1 of 3 *A. flavus* strains produced aflatoxins B_1_ and B_2_ without G_1_ and G_2_. Although the sample contamination ratios were high (≥95.6%), there was no significant difference (*χ* ^2^ = 1.05, *P* = 1.0) among the samples from 3 regions, which demonstrates the prevalent fungal contamination in the herbal medicines.

**Conclusion:**

The prevalent contamination phenomenon of fungi and high potential risk of sterigmatocystin and ochratoxin A were observed in 45 medicinal herbs collected from China.

**Electronic supplementary material:**

The online version of this article (doi:10.1186/s13020-016-0124-7) contains supplementary material, which is available to authorized users.

## Background

With the popular and extensive use of medicinal herbs all over the world, safety issues related to the contamination with microbial organisms has become a major concern [1–4]. Most of fungi are toxigenic in nature, and some other non-toxigenic species may impart a mouldy odour and taste [5]. In the pre-harvest stage, medicinal herbs are susceptible to indigenous fungi in the soil where they were grown. The dried part of medicinal herbs may be exposed to fungal contamination during post-harvest. Different taxonomic groups of fungi were detected in medicinal plant samples collected from different regions, suggesting *Aspergillus* and *Penicillium* groups as the most predominant genera [6–8]. Many species of *Aspergillus* and *Penicillium* genera are known mycotoxin-producers, which may pose a great threat to public health [5].

Mycotoxigenic fungi could produce a wide variety of mycotoxins. Aflatoxins (B_1_, B_2_, G_1_, and G_2_) are a family of structurally related toxic secondary metabolites which mainly produced by certain strains of *Aspergillus flavus* (*A. flavus*) and *Aspergillus Parasiticus* (*A. parasiticus*) [9, 10]. Aflatoxin B_1_ (AFB_1_) was classified as a Group I carcinogen by the World Health Organization for Research on Cancer in 1993 [11]. Sterigmatocystin (ST), the stable intermediate in the final steps of aflatoxin biosynthesis in the aflatoxin-producing fungi *A. flavus* and *A. parasiticus*, was proven to be another carcinogenic mycotoxin [12]. Some certain stains, e.g., *A. versicolor*, *A. sydowi*, *A. nidulans*, *Bipolaris*, *Chaetomium* and *Emericella* spp. could also produce ST [13–16]. Produced by *P. verrucosum*, *P. nordicum* and *A. carbonarius* [17–19], another mycotoxin ochratoxin A (OTA) could cause a series of adverse effects in animals and humans, including teratogenicity, immunotoxicity, genotoxicity and mutagenicity [20–22].

This study aimed to investigate the presence of fungi on the surface of 45 medicinal herbs samples of fifteen herbs collected from Hunan, Hubei and Guangxi Province, China, by the characterization of morphology and ITS sequencing, followed by analysis of mycotoxigenic potential of isolated fungi using LC-MS/MS for the measurement of AFB_1_, AFB_2_, AFG_1_, AFG_2_, ST and OTA.

## Methods

### Chemicals and reagents

Standard solutions of AFB_1_, AFB_2_, AFG_1_ and AFG_2_, and OTA and ST powder standards were purchased from Supelco Sigma-Aldrich (St Louis, MO, USA). Potato dextrose agar (PDA) with chloramphenicol (0.1 g/L) was obtained from Huan-Kai (Guangzhou, China). LC-grade acetonitrile and methanol were bought from Merck (Darmstadt, Germany). Ultra-pure water was obtained from a Millipore Q system (Millipore, France). The other reagents were of analytical grade and bought from local producers.

### Samples collection

Fifteen kinds of most commonly-used medicinal herbs (Table 1) were chosen for analysis. During July and August of 2010, about 30 g of each kind of medicinal herb were purchased from a random herbal medicine store in Hunan, Hubei and Guangxi province (China), respectively. The herbs were authenticated by an experienced pharmacist for traditional Chinese medicine according to Chinese Pharmacopeia [23]. Each sample was instantly put into a sterile polythene bag, sealed properly, shipped to the laboratory and stored at 4 °C prior to use. The detail information of each sample was given as Additional file 1: Table S1. The samples were processed as soon as possible to avoid second contamination.

**Table 1:** Number of different fungal species isolated from different medicinal herbs

Name of Samples	No. of samples	Fungal species^a^									
		*A. flavus*	*A. versicolor*	*A. aculeatus*	Other *Asper*-*gillus* spp.	*Euro*-*tium* spp.	*Penic*-*illium* spp.	*Clados*-*porium* spp	*Fusa*-*rium* spp.	Others	Total
Bulbus *Fritillariae cirrhosae*	3	-^b^	–	–	–	1	2	–	–	1	4
Cortex *Eucommiae*	3	–	–	–	–	–	2	–	–	1	3
Cortex *Magnoliae officinalis*	3	–	–	–	0	1	–	–	–	1	2
Flos *Carthami*	3	2	–	–	2	1	2	–	–	6	13
Flos *Lonicerae japonicae*	3	–	–	–	–	1	2	–	–	0	3
Fructus *Lycii*	3	1	1	–	1	–	–	–	–	4	7
Herba *Andrographis*	3	–	1	–	3	4	5	2	–	3	18
Radix *Angelicae sinensis*	3	–	–	1	1	1	3	–	1	–	7
Radix *Astragali*	3	–	–	–	3	–	2	3	–	4	12
Radix *Codcnopsitis Pilosulas*	3	–	–	1	–	–	5	–	1	1	8
Radix *et* Rhizoma *Glycyrrhizae*	3	–	2	1	2	–	2	1	1	6	15
Radix *Notoginseng*	3	–	1	–	1	–	2	–	–	2	6
Radix *Panacis quinquefolii*	3	–	–	–	–	–	3	2	–	6	11
Radix *Pseudostellariae*	3	–	1	–	–	–	3	1	–	–	5
Semen *Armeniacae amarae*	3	–	2	–	2	5	2	1	–	–	12
Total	45	3	8	3	14	14	35	10	3	36	126

^a^The strains were deposited in Research Centre of Chinese Herbal Resource Science and Engineering, Guangzhou University of Chinese Medicine, Guangzhou, China

^b^Not found

### Isolation of the fungi

Five grams of each sample (2.5 g was used for flower samples) was mixed with 30 mL 0.1% Tween-20 and vortexed (Vortex-5, Qilinbeier, China) for 3 min. Then the mixture was filtrated through a disposable syringe with sterile cotton and the filtrate was centrifuged (3-18 K, Satorius, Germany) at 2600×*g* for 10 min. After dissolving the pellet in 300 µL sterile 40% glycerol, serial decimal dilutions were performed. For incubation, 100 μL aliquots of each dilution were plated in duplicate onto PDA containing chloramphenicol (0.1 g/L), which were cultured at 28 °C for 7–10 days. Meanwhile, a sample without medicinal herbs was prepared in parallel and used as the negative control. The fungal colonies were then transferred to fresh PDA plates to obtain pure cultures, with 0.1% Tween-20 as negative control.

### Morphological observation

The purified isolates were cultured with 1 or 3 point inoculations for 7 days on PDA at 28 °C. When the colony characteristics and pigment production were noted, the conidia and conidial head were observed microscopically (Smart, Optec, China) for morphological identification by lactophenol cotton blue stain. Taxonomic identification results were classified based on Manual of fungal Identification into Aspergillus, Penicillium and Fusicladium *etc* [24–27].

### Molecular identification by ITS primers

After the pure isolates were grown on PDA at 28 °C for 7 days, the mycelia were harvested. DNA extraction was performed using the Lysis Buffer for Microorganism to Direct PCR kit (Takara, Dalian, China**)** according to the manufacturer’s instructions. Fungal mycelium was added to 50 µL lysis buffer and incubated at 80 °C for 15 min. After centrifugation, the supernatant was collected and used as the polymerase chain reaction (PCR) template. Positive control was performed with the standard strain *Aspergillus flavus* NRRL3357.

PCR amplification was performed in a 50 µL reaction prepared by mixing 25 µL 2× Power *Pfu* PCR Mixture (Bioteke, Beijing, China), 2.5 µL oligonucleotide primers (10 µmol/L) and 5 µL DNA template. Two pairs of primers (Table 2) were used to amplify ITS1, 5.8S and ITS2 in rDNA regions. The novel primers of Wen1-F and Wen1-R were designed according to the ITS sequences of *Penicillium expansu* (AJ005676.1) and *P. janthinellum* (GU565108.1). The amplification program was as follows: pre-denaturation at 95 °C for 5 min; 35 cycles of 30 s at 95 °C, 30 s at 55 °C and 1 min at 72 °C; and final extension at 72 °C for 10 min. The sequence analysis was performed at Huada Co. Ltd. (Guangzhou, China) and the sequencing results were analysed with the BLAST program of the National Centre for Biotechnology Information (NCBI) by searching NCBI nucleotide database (RRID:SCR_004860) for identification of the genus and species of the isolates.

**Table 2:** Oligonucleotide primers used for molecular identification

Primer name	Primer sequence	Amplification product (bp)	Annotation	Gene targeted
Wen1-F	5′–TCCAACCTCCCACCCGTGTTTA–3′	400	This study	ITS1-5.8S-ITS2
Wen1-R	5′–AAGCCCCTACGCTCGAGGA–3′			
ITS1	5′–TCCGTAGGTGAACCTGCG–3′	500 ~ 700	[32]	
ITS4	5′–TCCTCCGCTTATTGATATGC–3′			

### Mycotoxin-producing potential analysis

The assay was to aim to determine AFB_1_, AFB_2_, AFG_1_, AFG_2_, OTA and ST. Therefore, only the potential producer,according to literatures, were screened and the other 16 strains were excluded. Among the total isolates obtained from the samples, 110 strains potentially producing 6 mycotoxins were grown in Sabouraud dextrose medium (SD) at 28 °C for 10 days. Then 5 mL the culture broth was extracted with ethyl acetate followed by dichloromethane [28] and the organic layers were combined and then evaporated to dryness. After that, the residues were dissolved in 1.5 mL ethanol and injected into liquid chromatography-tandem mass spectrometry (LC–MS/MS) for determination of AFB_1_, AFB_2_, AFG_1_, AFG_2_, OTA and ST. Meanwhile, the standard strain *Aspergillus flavus* NRRL3357 were used as positive control.

**Fig. 1:**
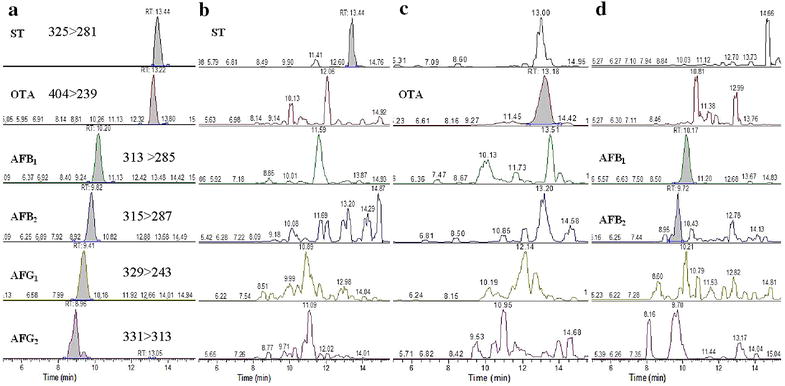
SRM Chromatograms of 6 mycotoxins in standard solution (**a**), *A. versicolor* isolated from Herba *Andrographi*s (**b**), *A. aculeatus* isolated from Radix *Angelicae sinensis* (**c**) and *A. flavus* isolated from *Fructus lycii* (**d**)

Liquid chromatography separation of 10 µL sample was performed on a Hypersil GOLD C_18_ (100 × 2.1 mm, 3 µm) column. The mobile phase consisted of (A) water containing 4 m mol/L NH_4_Ac–0.1% HCOOH and (B) methanol and the flow rate was 300 μL/min. A gradient elution program was applied: 0‒10 min, 20‒85% B; 10‒15 min, 85‒100% B; 15‒20 min, 100% B. The mass spectrometer was operated in the ESI^+^ mode using selective reaction monitoring (SRM). High-purity nitrogen was used as the drying and ionisation gas. Argon was used as the collision gas for collision-induced dissociation. The capillary voltage was set at 3.50 kV and the capillary temperature was 350 °C. The SRM transitions used to detect mycotoxins were listed in Table 3 and chromatograms of 6 mycotoxins in standard solution and representative samples were showed in the Fig. 1.

**Table 3:** The ESI-MS/MS parameters, retention time, SRM transitions and LOD for 6 mycotoxins

Mycotoxin	RT (min)	Precursor ion (m/z)	Product ions (m/z)	Collision energy (eV)	LOD (ng/L)
Aflatoxin B_1_	10.20	313 [M + H]^+^	285/241	23/37	5.20
Aflatoxin B_2_	9.82	315 [M + H]^+^	287/259	27/31	6.30
Aflatoxin G_1_	9.41	329 [M + H]^+^	243/200	27/45	10.60
Aflatoxin G_2_	8.96	331 [M + H]^+^	313/245	26/30	5.80
Ochratoxin A	13.22	404 [M + H]^+^	239/358	25/15	25.00
Sterigmatocystin	13.44	325 [M + H]^+^	281/310	36/25	1.56

### Statistical analysis

Software RStudio (R version 3.2.2) [29] was used for the data analysis, where the comparison of fungal contamination ratios was performed by Pearson’s Chi squared test with simulated *P* value (based on 2000 replicates).

## Results and discussion

### Fungal contamination

The association between fungal species and herbal medicines is not fully understood due to the complicated contamination causes including extrinsic (environmental and geographical) and intrinsic (constituents of each herbal species) factors [30, 31]. Forty-five samples of 15 common medicinal herbs were investigated in this study to reveal the main contaminating fungi and provide some relevant references for quality control on medicinal herbs in China.

In this study, morphological analysis as well as molecular identification using ITS sequencing were applied to analyse fungal diversity. As not all the strains could be amplified by the primer pair ITS1/ITS4 [32], a novel primer pairs Wen1F/Wen1R were designed for the ITS regions of the fungi by Primer-BLAST of NCBI (Table 2). A total of 126 isolated strains were successfully amplified. It is notable that the primers of Wen1F/Wen1R tended to be biased towards the amplification of *Aspergillus* and *Penicillium*, which agreed with the view on the potential primers bias during PCR in fungal diversity exploring [33].

**Fig. 2:**
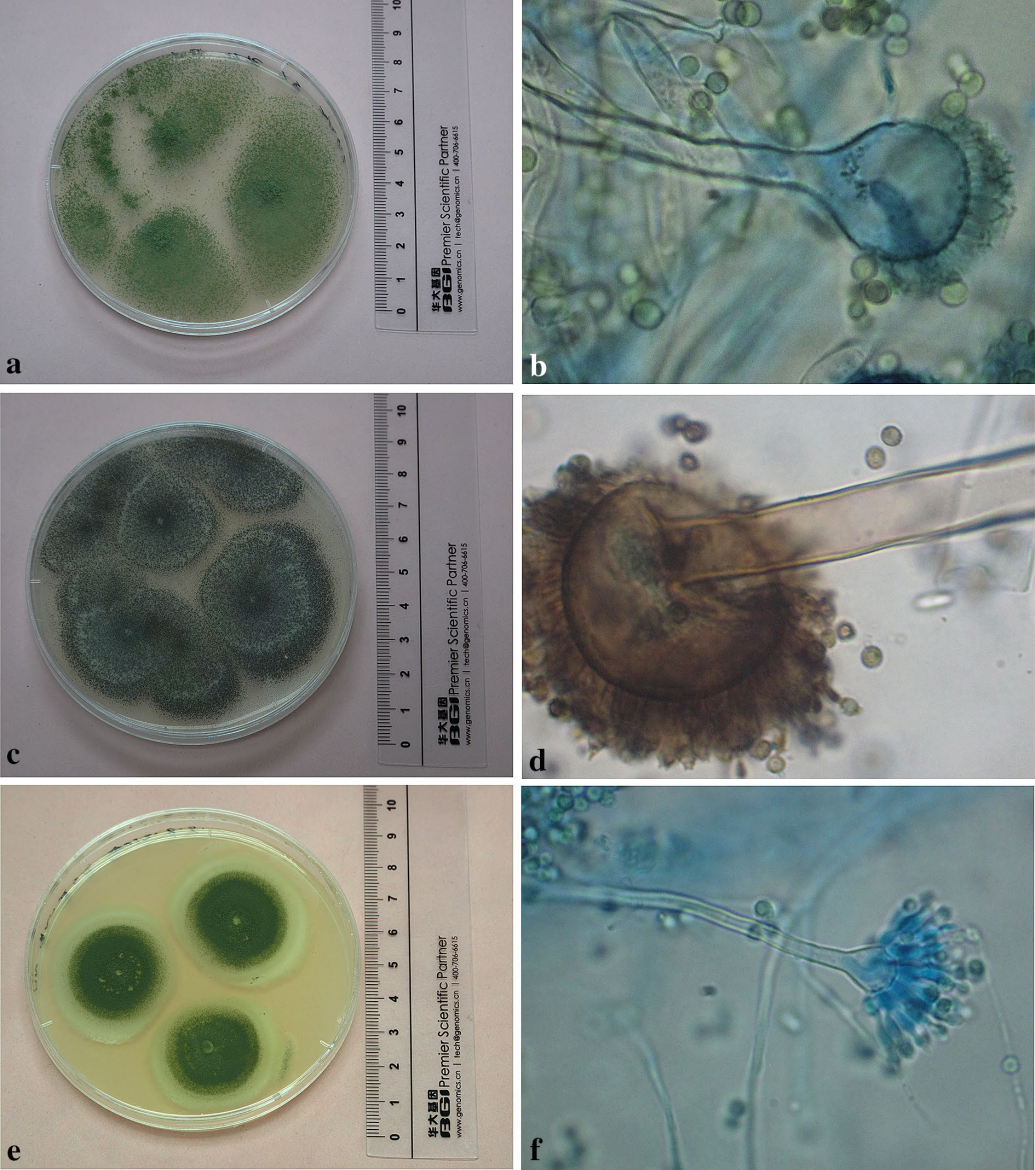
Colony morphologies and microscopic characteristic of some fungal isolates. **a**, **b** *A. flavus*; **c**, **d** *A. aculeatus*; **e**, **f** *A. versicolor*. **a**, **c** and **e** Colonies after incubation for 7 days at 25 °C on PDA. **b**, **d** and **f** conidial heads and conidiophores. Magnification 10 × 100

As a result, 126 strains were isolated (Table 1) illustrating the two main genera identified were *Aspergillus* (28 isolates) and *Penicillium* (35 isolates). Among of 28 *Aspergillus* recovered, *A. versicolor* (8 strains) was the dominant species, followed by the *A. fumigatus* (4 strains), *A. aculeatus* (3 strains) and *A. flavus* (3 strains). Other members of the *Aspergillus* group were detected at lower level. The colonies and microscopic morphologies of *A. flavus*, *A. aculeatus* and *A. versicolor* isolated from Fructus *Lycii*, Radix *Angelicae Sinensis* and Radix *et* Rhizoma *Glycyrrhizae*, respectively, were shown in Fig. 2.

Although the average of fungal contamination ratio (95.6%) are extremely high in 45 samples collected from 3 places (93.3, 93.3, 100% for samples from Hubei, Hunan and Guangxi, respectively), Pearson’s Chi squared test indicated there is no significant difference among 3 groups by *P* = 1.0. This further proved the prevalent fungal contamination phenomenon across the collected herbal medicine and should rise our attention.

Overall, the observation of *Aspergillus* and *Penicillium* spp. as the most frequently contaminant was consistent with previous reports. Efuntoye [30] found that *A. niger*, *A. flavus*, *F. moniliforme*, *Trichoderma viride*, *P. expansum* and *Mucor fragilis* were the dominant species in sundried herbs. Roy et al. [34] reported that 52% of 152 samples were contaminated with species from the *Aspergillus* genus, while Halt [35] found that the most predominant fungi detected in 62 samples of medicinal plant material and 11 herbal tea samples were *Aspergillus* and *Penicillium*.

In comparison, fungi from other genera including *Eurotium*, *Cladosporium* was detected at a low incidence in this study, which was also consistent with the study from Song et al. [3]. *Cladosporium* spp. is the common and widespread fungi on land and in air [36, 37]. Although there are no publications regarding its ability of producing toxin, they do produce odours likely relating to some volatile organic compounds.

### Mycotoxigenic potentials of the fungal isolates

As documented in Table 3, the LC-MS/MS method showed an outstanding sensitive, with the limit of detection (LOD) from 1.56 to 25.00 ng/L determined in 3 times of the ratio of signal to noise (S/N). The data on the mycotoxin-producing potentials of the fungal isolates were presented in Table 4. Of the 3 *A. flavus* isolates, only 1 strain from Fructus *Lycii* produced AFB_1_ and AFB_2_ but not AFG_1_ and AFG_2_. The inability of the other 2 strains to produce aflatoxins might be a result of some mutation in the biosynthetic gene cluster of aflatoxins [38].

**Table 4:** Toxigenic potentials of the isolates from 45 samples of medicinal herbs

Fungi	Source	No. of strains isolated	No. of positive strains	Toxin production^a^
*A. flavus*	Flos *Carthami*	2	0	–^b^
*A. flavus*	Fructus *Lycii*	1	1	AFB_1_, AFB_2_
*A. versicolor*	Radix *et* Rhizoma *Glycyrrhizae*	2	2	ST
*A. versicolor*	Semen *Armeniacae amarae*	2	1	ST
*A. versicolor*	Herba *Andrographis*	1	1	ST
*A. versicolor*	Radix *Pseudostellariae*	1	1	ST
*A. versicolor*	Fructus *Lycii*	1	1	ST
*A. versicolor*	Radix *Notoginseng*	1	0	–
*A. aculeatus*	Radix *et* Rhizoma *Glycyrrhizae*	1	1	OTA
*A. aculeatus*	Radix *Codcnopsitis pilosulas*	1	1	OTA
*A. aculeatus*	Radix *Angelicae sinensis*	1	1	OTA

^a^ Mycotoxins determined including AFB_1_, AFB_2_, AFG_1_, AFG_2_, OTA and ST

^b^ Below the detection limits

Another mycotoxin ST, which was overlooked in many reports except a study of mycotoxins screening in medicinal herbs [39], presented in 6 of the 8 *A. versicolor* isolates by the analysis of LC-MS/MS. To evaluate human exposure to this mycotoxin and more importantly, monitor medicinal herbs for existing or future legal compliance, suitable and simple analytical procedures are necessary to precisely analyse it and its contamination phenomenon in these samples even all the medicinal herbs.

Consistent to Blank et al. [40], all the *A. aculeatus* strains isolated in this study produced OTA. Although it has been reported that a number of *Penicillium* strains are OTA producers [17, 19, 41], none of 35 *Penicillium* strains in this study produced OTA, which has to be further confirmed. Moreover, the contaminant *A. fumigatus* still should not be neglected, even though neither the *A. fumigatus*, *Cladosporium* spp. strains nor the *Penicillium* spp. produced detectable mycotoxins. Because *A. fumigatus* is thermos-tolerant and has the ability to excrete hydrolytic extracellular enzymes that consequently allow opportunistic colonisation in lung tissue [42].

## Conclusion

The prevalent contamination phenomenon of fungi observed in 45 medicinal herbs collected from China and the mycotoxigenic potential of some fungal isolates suggested appropriate procedures should be engaged to protect medicinal herbs from being contaminated.

## Additional file

**Additional file 1: Table S1.** Samples collection information and depositing numbers.

## Abbreviations

AFB_1_: aflatoxin B_1_
AFB_2_: aflatoxin B_2_
AFG_1_: aflatoxin G_1_
AFG_2_: aflatoxin G_2_
ITS: internal transcribed spacer
LC-MS/MS: liquid chromatography-tandem mass spectrometry
LOD: limit of detection
NCBI: National Centre for Biotechnology Information
OTA: ochratoxin A
PCR: polymerase chain reaction
PDA: potato dextrose agar
SD: sabouraud dextrose medium
SRM: selective reaction monitoring
ST: sterigmatocystin
