# Supplementary material for: An investigation of fungal contamination on the surface of medicinal herbs in China
Source: Chin Med. 2017 Jan 3;12:2. doi: 10.1186/s13020-016-0124-7 (PMC5209813; doi:10.1186/s13020-016-0124-7)
Supplement: Supplementary file 1 — Additional file 1: Table S1. Samples collection information and depositing numbers. [file 13020_2016_124_MOESM1_ESM.docx]

**Table S1 Samples collection information and depositing numbers**

| Depositing Numbers | Sample names | Collection regions | Collection dates |
| --- | --- | --- | --- |
| CMB_HN_20100721 | Bulbus *Fritillariae Cirrhosae* | Hunan Province, Chna | July 21, 2010 |
| DZ_HN_20100721 | Cortex *Eucommiae* | Hunan Province, Chna | July 21, 2010 |
| HP_HN_20100721 | Cortex *Magnoliae officinalis* | Hunan Province, Chna | July 21, 2010 |
| HH_HN_20100721 | Flos *Carthami* | Hunan Province, Chna | July 21, 2010 |
| JYH_HN_20100721 | Flos *Lonicerae japonicae* | Hunan Province, Chna | July 21, 2010 |
| GQ_HN_20100721 | Fructus *Lycii* | Hunan Province, Chna | July 21, 2010 |
| CXL_HN_20100721 | Herba *Andrographis* | Hunan Province, Chna | July 21, 2010 |
| DG_HN_20100721 | Radix *Angelicae Sinensis* | Hunan Province, Chna | July 21, 2010 |
| HQ_HN_20100721 | Radix *Astragali* | Hunan Province, Chna | July 21, 2010 |
| DS_HN_20100721 | Radix *Codonopsis Pilosulas* | Hunan Province, Chna | July 21, 2010 |
| GC_HN_20100721 | Radix *et* Rhizoma *Glycyrrhizae* | Hunan Province, Chna | July 21, 2010 |
| SQ_HN_20100721 | Radix *Notoginseng* | Hunan Province, Chna | July 21, 2010 |
| XYS_HN_20100721 | Radix *Panacis Quinquefolii* | Hunan Province, Chna | July 21, 2010 |
| TZS_HN_20100721 | Radix *Pseudostellariae* | Hunan Province, Chna | July 21, 2010 |
| KXR_HN_20100721 | Semen *Armeniacae Amarae* | Hunan Province, Chna | July 21, 2010 |
| CMB_HB_20100709 | Bulbus *Fritillariae Cirrhosae* | Hubei Province, Chna | July 9, 2010 |
| DZ_HB_20100709 | Cortex *Eucommiae* | Hubei Province, Chna | July 9, 2010 |
| HP_HB_20100709 | Cortex *Magnoliae officinalis* | Hubei Province, Chna | July 9, 2010 |
| HH_HB_20100709 | Flos *Carthami* | Hubei Province, Chna | July 9, 2010 |
| JYH_HB_20100709 | Flos *Lonicerae japonicae* | Hubei Province, Chna | July 9, 2010 |
| GQ_HB_20100709 | Fructus *Lycii* | Hubei Province, Chna | July 9, 2010 |
| CXL_HB_20100709 | Herba *Andrographis* | Hubei Province, Chna | July 9, 2010 |
| DG_HB_20100709 | Radix *Angelicae Sinensis* | Hubei Province, Chna | July 9, 2010 |
| HQ_HB_20100709 | Radix *Astragali* | Hubei Province, Chna | July 9, 2010 |
| DS_HB_20100709 | Radix *Codonopsis Pilosulas* | Hubei Province, Chna | July 9, 2010 |
| GC_HB_20100709 | Radix *et* Rhizoma *Glycyrrhizae* | Hubei Province, Chna | July 9, 2010 |
| SQ_HB_20100709 | Radix *Notoginseng* | Hubei Province, Chna | July 9, 2010 |
| XYS_HB_20100709 | Radix *Panacis Quinquefolii* | Hubei Province, Chna | July 9, 2010 |
| TZS_HB_20100709 | Radix *Pseudostellariae* | Hubei Province, Chna | July 9, 2010 |
| KXR_HB_20100709 | Semen *Armeniacae Amarae* | Hubei Province, Chna | July 9, 2010 |
| CMB_GX_20100803 | Bulbus *Fritillariae Cirrhosae* | Guangxi Province, Chna | August 3, 2010 |
| DZ_GX_20100803 | Cortex *Eucommiae* | Guangxi Province, Chna | August 3, 2010 |
| HP_GX_20100803 | Cortex *Magnoliae officinalis* | Guangxi Province, Chna | August 3, 2010 |
| HH_GX_20100803 | Flos *Carthami* | Guangxi Province, Chna | August 3, 2010 |
| JYH_GX_20100803 | Flos *Lonicerae japonicae* | Guangxi Province, Chna | August 3, 2010 |
| GQ_GX_20100803 | Fructus *Lycii* | Guangxi Province, Chna | August 3, 2010 |
| CXL_GX_20100803 | Herba *Andrographis* | Guangxi Province, Chna | August 3, 2010 |
| DG_GX_20100803 | Radix *Angelicae Sinensis* | Guangxi Province, Chna | August 3, 2010 |
| HQ_GX_20100803 | Radix *Astragali* | Guangxi Province, Chna | August 3, 2010 |
| DS_GX_20100803 | Radix *Codonopsis Pilosulas* | Guangxi Province, Chna | August 3, 2010 |
| GC_GX_20100803 | Radix *et* Rhizoma *Glycyrrhizae* | Guangxi Province, Chna | August 3, 2010 |
| SQ_GX_20100803 | Radix *Notoginseng* | Guangxi Province, Chna | August 3, 2010 |
| XYS_GX_20100803 | Radix *Panacis Quinquefolii* | Guangxi Province, Chna | August 3, 2010 |
| TZS_GX_20100803 | Radix *Pseudostellariae* | Guangxi Province, Chna | August 3, 2010 |
| KXR_GX_20100803 | Semen *Armeniacae Amarae* | Guangxi Province, Chna | August 3, 2010 |
